# Reduced Levels of the Synaptic Functional Regulator FMRP in Dentate Gyrus of the Aging Sprague-Dawley Rat

**DOI:** 10.3389/fnagi.2017.00384

**Published:** 2017-11-23

**Authors:** Roman Smidak, Fernando J. Sialana, Martina Kristofova, Tamara Stojanovic, Dragana Rajcic, Jovana Malikovic, Daniel D. Feyissa, Volker Korz, Harald Hoeger, Judit Wackerlig, Diana Mechtcheriakova, Gert Lubec

**Affiliations:** ^1^Department of Pharmaceutical Chemistry, Faculty of Life Sciences, University of Vienna, Vienna, Austria; ^2^Core Unit of Biomedical Research, Division of Laboratory Animal Science and Genetics, Medical University of Vienna, Vienna, Austria; ^3^Department of Pathophysiology and Allergy Research, Medical University of Vienna, Vienna, Austria; ^4^Neuroproteomics, Paracelsus Private Medical University, Salzburg, Austria

**Keywords:** Fragile X syndrome, Fragile X mental retardation protein, brain aging, dentate gyrus, proteomics, RNA-binding protein

## Abstract

Fragile X mental retardation protein (FMRP) encoded by Fragile X mental retardation 1 (*FMR1*) gene is a RNA-binding regulator of mRNA translation, transport and stability with multiple targets responsible for proper synaptic function. Epigenetic silencing of *FMR1* gene expression leads to the development of Fragile X syndrome (FXS) that is characterized by intellectual disability and other behavioral problems including autism. In the rat FXS model, the lack of FMRP caused a deficit in hippocampal-dependent memory. However, the hippocampal changes of FMRP in aging rats are not fully elucidated. The current study addresses the changes in FMRP levels in dentate gyrus (DG) from young (17 weeks) and aging (22 months) Sprague – Dawley rats. The aging animal group showed significant decline in spatial reference memory. Protein samples from five rats per each group were analyzed by quantitative proteomic analysis resulting in 153 significantly changed proteins. FMRP showed significant reduction in aging animals which was confirmed by immunoblotting and immunofluorescence microscopy. Furthermore, bioinformatic analysis of the differential protein dataset revealed several functionally related protein groups with individual interactions with FMRP. These include high representation of the RNA translation and processing machinery connected to FMRP and other RNA-binding regulators including CAPRIN1, the members of Pumilio (PUM) and CUG-BP, Elav-like (CELF) family, and YTH N(6)-methyladenosine RNA-binding proteins (YTHDF). The results of the current study point to the important role of FMRP and regulation of RNA processing in the rat DG and memory decline during the aging process.

## Introduction

The Fragile X mental retardation protein (FMRP) is a RNA-binding regulator encoded by Fragile X mental retardation 1 (*FMR1*) gene. This protein is ubiquitously produced in mammalian tissues with high levels in brain (Tamanini et al., 1997), and localized predominantly in cytosolic light and heavy membranes, and nucleus (Taha et al., 2014). It contains two K-homology domains (KH1 and KH2) and a C-terminal RGG box, involved in RNA-binding, together with nuclear localization and nuclear export signal (Adinolfi et al., 1999; Valverde et al., 2007). These structural features suggest the molecular functions of FMRP which have been associated with mRNA stability (Zalfa et al., 2007), nuclear – cytoplasmic shuttling of mRNA (Eberhart et al., 1996) and repression of mRNA translation (Jin and Warren, 2000; Li et al., 2001; Kalidas and Smith, 2003). The total number of FMRP functions *in vivo* is not known and a multitude of FMRP targets was proposed based on large-scale analyses (Ascano et al., 2012; Ouwenga and Dougherty, 2015).

Fragile X mental retardation protein has been implicated as a regulator of synaptic plasticity and thereby learning and memory (reviewed in Bostrom et al., 2016). The functions of FMRP in nervous system have been related to the control of synaptic protein synthesis in response to neurotransmitter activation and affecting the formation of axonal and dendritic structures (Feng et al., 1997; Greenough et al., 2001; Weiler et al., 2004; Zimmer et al., 2017). The FMRP-mediated regulation of mRNA translation has been also directly linked to neurotransmitter receptor systems involved in synaptic plasticity. As an example, it was shown that the activation of metabotropic glutamate receptors (mGluR) affects FMRP localization in dendrites and synapses (Antar et al., 2004), FMRP deficiency leads to excessive mGluR5- and local protein synthesis-dependent internalization of AMPA receptors (Nakamoto et al., 2007) and that the expression of the NMDA receptor subunit NR2A is regulated by FMRP-associated microRNAs (Edbauer et al., 2010).

Impairment of *FMR1* gene expression due to amplification of CGG repeat in its 5′-UTR and hypermethylation leads to several syndromes. The full mutation allele (>200 CGG repeats) results in lack of FMRP and development of Fragile X syndrome (FXS) that is characterized by cognitive disabilities and behavioral disorders (Wang et al., 2012). The mouse and rat *FMR1* knock-out (KO) models have been extensively used to study behavioral deficits, and alterations in brain physiology and development associated with FXR (Till et al., 2012, 2015). Interestingly, a recent quantitative proteomic study of *FMR1* KO mouse revealed a significant overlap of a dysregulated proteome between the FXR condition and normal aging (Tang et al., 2015) and the FMRP levels were found to be decreased in aged mouse brain (Singh et al., 2007; Singh and Prasad, 2008).

The information on aging-dependent changes of FMRP in rat brain and specific brain subareas is limited. The dentate gyrus (DG) is a hippocampal subregion that plays a critical role in information processing including spatial memory (Jonas and Lisman, 2014; Bott et al., 2016) and the aging-related functional alterations in this region were previously described for rats, mice, and other mammalian models (Lister and Barnes, 2009). The results obtained from *FMR1* KO mouse models indicate an important function of FMRP in physiology, structure and connectivity of DG, and the related behavior (Bostrom et al., 2016; Lai et al., 2016; Scharkowski et al., 2017). Previous studies on rats showed deficits in hippocampal-dependent memory in a *FMR1* KO model (Till et al., 2015) and behavior-induced changes of FMRP level in DG (Irwin et al., 2005). However, validated information on FMRP levels in DG in rat during normal aging is missing. In the current study, FMRP levels in DG were compared between the young (17 weeks) and aging (22 months) Sprague-Dawley rats. In the initial hole-board test the aging group showed a significant decline in spatial reference memory. Protein samples of DG from five rats per each group were then analyzed by quantitative proteomic analysis resulting in 153 significantly changed proteins. FMRP showed significant reduction in aging animals which was confirmed by immunoblotting and immunofluorescence microscopy. Furthermore, bioinformatic analysis of the differential protein dataset revealed several functionally related protein groups with individual interactions with FMRP. These included high representation of RNA translation and processing machinery connected to FMRP and other RNA-binding regulators. The results of the current study point to an important role of FMRP and the regulation of RNA processing in the rat DG related to memory decline during the aging process.

## Materials and Methods

### Animals

Animals were bred and maintained in the Core Unit of Biomedical Research, Division of Laboratory Animal Science and Genetics, Medical University of Vienna. The animals lived in a separate experimental room 1 week before and throughout the experiment. Rats were housed individually in standard Makrolon cages filled with autoclaved woodchips (temperature: 22 ± 2°C; humidity: 55 ± 5%; 12 h artificial light/12 h dark cycle: light on at 7:00 am). The study was carried out in accordance the recommendation of the European Communities Council Directive of 24 November 1986 (86/609/EEC) evaluated by the ethics committee of the Medical University of Vienna, Vienna, Austria. The protocol was approved by Federal Ministry of Education, Science and Culture, Austria.

### Hole-Board Memory Test

The hole-board consisted of a 1 m × 1 m board made of black plastic surrounded by translucent plexiglass walls. Each side of the wall bears proximal spatial cues. Distal cues were provided by room structures visible outside the board. Four out of sixteen regularly arranged holes (diameter and depth 7 cm) were baited (dustless precision pellets, 45 mg, Bioserv^®^). The pattern of baited holes remained the same during the entire test. In order to avoid olfactory orientation pellets were also present in an area below the board. Rats were familiarized to the experimenter through 10 min handling sessions per day for 4 days prior to the experiment, followed by 2 days of habituation to the hole-board during which the animals were allowed to explore the maze for 15 min each day with access to food pellets. The weight of each rat was gradually reduced by food restriction to reach 85% of its free-feeding body weight before the start of the experiment. Tap water was given *ad libitum*. Thereafter the rats were trained for 3 days (five trials on day 1, four trials on day 2 and a retention trial at day 3). Every trial lasted for 120 s or until all four pellets were eaten. The apparatus was cleaned with 0.1% incidin between trials in order to remove the rat odor cues. The interval between two successive trials for an individual was 20 min. A camera mounted on the room ceiling recorded the performance of the rats in the maze. The visits of holes and removals of pellets were noted for each trial. In order to compare rats with similar levels of motivation, rats with less than 40 hole-visits in total over the 10 trials were excluded from the analysis. Reference memory errors were noted as the number of visits to the unbaited holes. The Reference Memory Index (RMI) was calculated using the formula: (first + revisits of baited holes)/total visits of all holes. All behavioral training/testing was performed during the light phase of the light–dark cycle. Rats were decapitated, the brains were rapidly removed and dissected on a Para Cooler (RWW Medizintechnik, Hallerndorf, Germany) at 4°C to obtain DG. The tissue was immediately stored at -80°C until further analysis.

### Protein Sample Preparation

All homogenization and centrifugation steps were carried out on ice and at 4°C, respectively. Brain tissues were homogenized in an ice-cold homogenization buffer [10 mM HEPES, pH 7.5, 300 mM sucrose, protease inhibitors (Roche)] using a Dounce homogenizer; the homogenate was centrifuged at 1000 × *g* for 10 min to remove cell debris and nuclei and the supernatant was collected. The pellet was resuspended again in the homogenization buffer and centrifuged at 1000 × *g* for 10 min. The pooled supernatants were then centrifuged at 100000 × *g* for 60 min to enrich membranes. The resulting membrane pellets were washed in 10 mM HEPES, pH 7.5, protease inhibitors (Roche) and solubilized in 50 mM TEAB buffer (Sigma–Aldrich), 7 M urea, 2 M thiourea, 4% CHAPS, 100 mM DTT and protease inhibitors (Roche). The protein concentration was determined by Pierce^TM^ 660 nm Protein Assay (Thermo Scientific).

### Quantitative LC–MS/MS Analysis

Protein samples were digested overnight with trypsin (Promega) using filter-aided sample preparation (FASP) (Wisniewski et al., 2009) with 50 μg of protein per one reaction. The tryptic peptides were desalted using reversed-phase C18 stage tips (Rappsilber et al., 2007) and reconstituted in 50 μL of 50 mM TEAB (Sigma–Aldrich, Hamburg, Germany). The samples (50 μg in 50 μL) were subsequently differentially labeled by TMT10plex^TM^ Isobaric Label Reagent Set (Thermo Scientific) according to the manufacturer’s instructions. Using an actual peptide concentration determined by Pierce^TM^ Quantitative Colorimetric Peptide Assay (Thermo Scientific) the samples were pooled by mixing an equal amount of labeled peptides from individual samples. The pooled sample was separated by reversed-phase liquid chromatography (RP-LC) at high pH as previously described (Gilar et al., 2005) resulting in 25 fractions. The peptide fractions were analyzed by liquid chromatography-tandem mass spectrometry (LC–MS/MS) with two technical replicates per sample using the LC separation procedure described previously (Smidak et al., 2016). MS analysis was performed by the Thermo Scientific^TM^ Q Exactive^TM^ Plus Orbitrap mass spectrometer (Thermo Scientific) in positive ion mode with full-scan MS in the range of *m/z* 375–1400 at the resolution of 35,000 (at m/z 200). MS/MS scans were acquired at the resolution of 70,000 (m/z 200) through HCD fragmentation of 15 most intense ions at 32% normalized collision energy with a fixed mass of 100 m/z. Dynamic exclusion was enabled for 30 s and for unassigned, +1 and ≥ +8 charges.

### Proteomic Data Analysis

MS raw data were searched against UniProtKB Rattus norvegicus complete proteome database (UP000002494, 31569 sequences, downloaded on December 2nd, 2016) using MASCOT 2.4 (MatrixScience) through Proteome Discoverer 2.1 platform (Thermo Scientific). The search criteria were as follows: trypsin with a maximum of two missing cleavage sites; fixed modification: carbamidomethylation (C); variable modification: oxidation (M); search mode: MS/MS ion search with decoy database search included; peptide mass tolerance ± 10 ppm; MS/MS mass tolerance ± 0.02 Da; and protein and peptide FDR 1%. Only proteins identified by two distinct peptides and one unique peptide were considered for the final dataset. The Reporter Ions Quantifier node in the Proteome Discoverer software was used to calculate the corresponding protein abundances based on the intensities of individual TMT reporter ion channels (126.13–131.14 m/z) corrected for reporter ions isotopic distribution. The protein abundances were normalized by the total intensity of each reporter in the PSMs (Peptide-Spectral-Matches) population. Differences in protein levels are expressed as log2-transformed normalized protein abundance ratio of aging versus young animal group evaluated by two-sided *t*-test using Perseus statistical package (version 1.3.0.4). *p*-Values < 0.05 with permutation-based FDR threshold 0.05 were used as cut-off for significance. Additional filtering for significantly changed proteins was applied based on log2 (protein ratio) threshold > |0.3|. Significantly changed proteins were analyzed for enrichment of GO annotations using ClueGO-Cytoscape platform (Bindea et al., 2009; Huntley et al., 2015) using GOA database 30.08.2017 and Benjamini–Hochberg correction (adjusted *p*-Values < 0.01). The lists of most representative pathways and biological functions/diseases, and protein interaction networks were generated by Ingenuity Pathway Analysis (IPA) software (Qiagen) using right-tailed Fisher’s exact test (*p* < 0.05). Functionally related protein clusters within the differential dataset were visualized by STRING v10.5^1^ and MCL algorithm with confidence threshold 0.5 and inflation parameter 3.

### Western Blot

The protein samples were separated by electrophoresis in 10% SDS-PAGE gel and transferred to PVDF membranes (GE Healthcare). The western blot procedure was performed as described previously (Shanmugasundaram et al., 2017) with minor modifications. Briefly, the membrane was blocked for 1 h in 5% non-fat milk, 1x TBS, 0.05% Tween-20 and probed with the corresponding primary and secondary antibodies. For detection of FMRP protein the membrane was incubated in 3% non-fat milk, 1x TBS, 0.1% Tween-20 with 1:1000 polyclonal mouse anti-FMRP antibody (MAB2160, Millipore) overnight at 4°C followed by incubation for 1 h at room temperature in 1:10000 HRP-conjugated anti-mouse secondary antibody (ab6728, Abcam). Actin as a loading control was detected by incubation in 3% non-fat dry milk, 1x TBS, 0.1% Tween-20 with 1:2000 anti-β-Actin antibody (4967S, Cell Signalling) overnight at 4°C followed by incubation for 1 h at room temperature in 1:10000 rabbit polyclonal antibody. The signals were quantified using ImageJ (NIH) software based on calculation of peak area and the intensity values for FMRP bands were normalized across the samples by actin control.

### Immunofluorescence Detection and Microscopy

For the analysis of FMRP expression eight young and eight aging rats were deeply anesthetized with isoflurane and transcardially perfused with 4% paraformaldehyde solution in 0.1 M sodium phosphate buffer, pH 7.4. Brains were gently removed and post-fixed in the same fixative solution for 20–24 h at 4°C. Each brain was rinsed well with phosphate buffered saline (PBS) to remove formaldehyde and incubated in 30% sucrose with 0.02% NaN_3_ at 4°C. Immediately prior to cutting, brains were embedded in O.C.T. Tissue-Tek (Sakura Finetek) and frozen at -20°C in a cryo chamber. Tissue was sectioned at 20 μm with a cryostat CryoStar NX50 (Thermo Scientific). Coronal cryo sections (Bregma 3.30–3.80 mm) were blocked in 5% normal goat serum (NGS) (G9023, Sigma–Aldrich), 0.3% Triton X-100 (T8787, Sigma–Aldrich), 2% BSA (23208, Thermo Scientific) in 0.1 M PBS 7.4 for 2 h at 22–24°C and incubated with primary antibody (mouse anti-FMRP 1:500 MAB2016, Merck) for 48 h at 4°C in 0.1 M PBS, 1% NGS, 0.1% Triton X-100, 0.1% BSA with continuous stirring. After three washing steps in 0.1 M PBS, sections were incubated with the secondary antibody (1:1000 AlexaFluor488 anti-mouse 4408S, Cell Signaling Technology) for 2 h at 22–24°C in 2% BSA. In addition, to remove lipofuscin-like autoimmunofluorescence, all sections were incubated in autoimmunofluorescence eliminator reagent (2160, Merck) for 5 min and then underwent six washing steps for 1 min each in 70% ethanol. DAPI staining was used to assess the gross cell morphology (D1306, Thermo Scientific). All sections were mounted with DAKO fluorescence mounting medium (Agilent Technologies) and analyzed by fluorescence microscopy. Immunoreactivity signal of FMRP in cells, processes and neutropil in the DG were acquired on a Zeiss LSM780 confocal microscope (Zeiss, Jena, Germany) using 20x magnification. The image analysis software ImageJ (NIH) calculated the percentage of FMRP-positive area per section by thresholding FMRP immunoreactivity above background levels. The cell body layer of DG granule cells and hilus were separately outlined as regions of interest (ROI) according to the DAPI signal in each slice. Immunofluorescence imaging and data analysis were duplicated, evaluating two brain slices per rat in total.

### Statistical Analysis

Data obtained from western blot and immunofluorescence microscopy was analyzed using Microsoft Excel 2013 or Prism 5 (GraphPad Software) and the difference between animal groups was evaluated by Student’s *t*-test with the significance cut-off *p* < 0.05. The behavioral data was analyzed with SPSS Version 22 software (IBM^®^ SPSS^®^ Statistics) using general linear model – repeated measures ANOVA for the differences over the entire training, or separately for days 1 and 2, and univariate ANOVA for the retention trial. The significance cut-off was set at *p* < 0.05. The values are expressed as mean ± SEM.

## Results

### Behavioral Testing

Behavioral testing was performed to assess hippocampal-dependent memory performance in the subarea of interest. The cohort of 18 young and 104 aging Sprague-Dawley rats were evaluated for spatial reference memory using the hole-board test. A significant effect of the entire training on memory performance (*p* = 0.032) was observed indicating that learning took place. Furthermore, the data showed a significant effect of the training-age factor interaction (*p* = 0.001) when the entire training was compared. The significant group differences were observed on day 2 (*p* = 0.001) and at the retention test at day 3 (*p* = 0.011) with the group of young animals performing significantly better than aging group (Supplementary Figure S1).

### Quantitative Proteomics Reveals Decreased Levels of FMRP

A quantitative MS-based approach was employed to identify proteins with significantly changed levels in the aging rats. The experimental design included two – young and aging – animal groups used in the previous behavioral test, five biological replicates per group. The membrane-enriched protein samples from DG were analyzed by LC–MS as TMT10plex labeling experiments in two sets representing two technical replicates per one biological replicate, i.e., two MS runs of each peptide fraction. Quantitative data for each individual are expressed as normalized protein abundance values for the corresponding TMT channel. A total number of 5832 proteins was identified in both groups, of which 153 were showing significantly different levels [*p* < 0.05, log2 (protein ratio) > |0.3|] between the groups. The complete list of significantly changed proteins as well as all identified proteins in the study is provided as supporting information (**Supplementary Table S1**).

**FIGURE 1:**
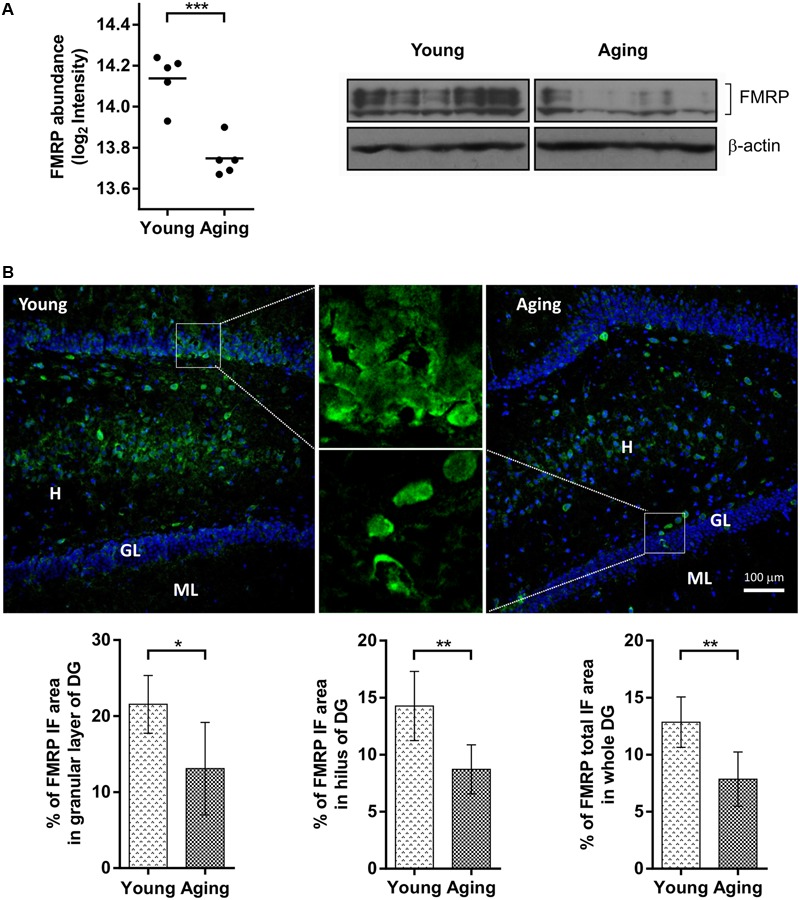
Comparison of FMRP levels between aging and young rats based on proteomics, immunoblotting and immunofluorescence (IF) microscopy. **(A)** The protein abundance levels of FMRP in young and aging rats obtained from a quantitative proteomic study (left) and immunoblot analysis (right). **(B)** Distribution of FMRP immunofluorescence analyzed in the hippocampal subareas of young and aging rats. Fluorescence images represent 10×, 20×, and 100× magnification of DG with FMRP labeled cells and neuropil in young and aging rats. Graphs represent percentage (%) of FMRP immunoreactivity in the granular layer (GL) and hilar (H) subareas of the DG area, as well as percentage (%) of total FMRP immunoreactivity throughout the whole DG. The differences were evaluated using Student’s *t*-test (*p* < 0.05). Values are expressed as mean ± SEM. ^∗^*p* < 0.05, ^∗∗^*p* < 0.01, ^∗∗∗^*p* < 0.001.

The FMRP was unambiguously identified in the proteomic samples as downregulated [log2 (protein abundance ratio) = -0.39, *p* = 0.0005] in the aging group (**Supplementary Table S1**) which was confirmed by western blotting (**Figure 1A**). The aging-dependent changes for FMRP in hippocampus were further analyzed by immunoflourescence microscopy with eight young and eight aging rats of the same behaviorally tested cohort. Consistently with general reduction in FMRP levels, there was a significant decrease (*p* = 0.002) in the total FMRP immunoreactivity per DG region in aging (7.86 ± 2.38) compared to young (12.85 ± 2.21) animals (**Figure 1B**). Furthermore, the comparison of individual DG subareas showed a significant decrease in FMRP labeling in aging rats in both, hilus (14.27 ± 3.03 vs. 8.72 ± 2.15, *p* = 0.002) and granular cell layer (21.55 ± 3.80 vs. 13.09 ± 6.08, *p* = 0.01).

### Bioinformatic Analysis of Proteomic Results

**FIGURE 2:**
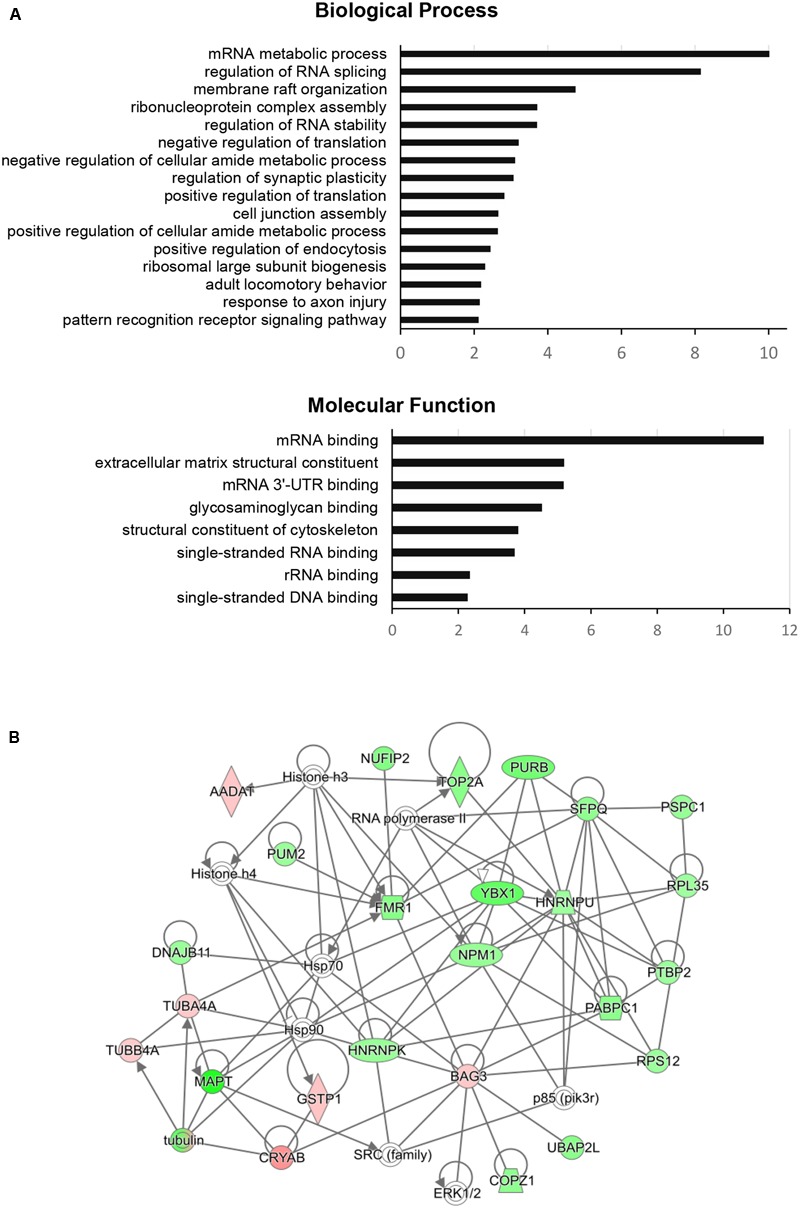
Functional enrichment analysis and FMRP (FMR1)-associated interaction network of significantly changed proteins in aging rats. **(A)** The GO categories of the most regulated biological processes and molecular functions associated with significantly changed proteins in the aging group. The GO enrichment analysis (Benjamini–Hochberg *p*-Values < 0.01) was performed using ClueGO-Cytoscape platform (Bindea et al., 2009; Huntley et al., 2015). **(B)** The protein interaction network of significantly changed proteins centered around FMRP retrieved from IPA analysis (QIAGEN). Green color represents downregulated proteins and red color represents upregulated proteins in the aging group. The protein nodes are connected based on RNA/DNA – interactions (directional lines) or protein – protein interactions (simple lines). Autoregulation is represented by loops. The gene abbreviations are given in the text and **Supplementary Table S1**.

To dissect the global proteomic changes identified in the current study functional enrichment analysis was applied to differential protein dataset. The biological processes and molecular functions that may be altered in aging animals were identified based on Gene Onthology (GO) enrichment (*p* < 0.01). The results for biological processes showed the overrepresentation of GO terms associated with RNA metabolism, regulation of RNA splicing and stability, membrane raft organization, cell junction assembly, regulation of endocytosis, and synaptic plasticity. The most significant molecular functions were related to RNA binding activity, glycosaminoglycan binding activity, structural constituents of extracellular matrix and cytoskeleton (**Figure 2A**). For more detailed annotation the data were submitted to IPA software (Qiagen) that infers activation states (z-score) of the representative pathways and biological functions (*p* < 0.05). The aging-dependent downregulation of EIF2 signaling (z-score = 2.646) followed by dysregulation in 14-3-3 mediated signaling, axonal guidance signaling and cell junction signaling were found as the most significant pathways. The analysis of biological functions showed coordinated expression changes leading to an increase of RNA splicing (z-score = 2.00), cell-to-cell contact (z-score = 2.05), a decrease in development of neurons (z-score = -2.13) and formation of microtubules (z-score = -2.14) in the aging group. The complete results obtained from GO enrichment and IPA analysis are provided as a supporting information (**Supplementary Table S2**).

The STRING network (STRING v10.5 database^2^) of significant proteins formed several clusters of closely related members associated with previously enriched processes. Most of the clusters were connected with each other reflecting the functional closeness of the identified machineries (Supplementary Figure S2). These include small groups of proteins involved in the formation of membrane structures and membrane vesicle trafficking (FLOT1, FLOT2, PASCIN2, BIN1, CLINT1), proteins associated with cell junctions (GJB2 GJB6, GJA1), and separate cluster of proteoglycans and proteoglycan-linked proteins (HAPLN1, VCAN, BCAN, ACAN, NCAN, TNC) linked to the group containing guanidine nucleotide binding (G) proteins (GNB4, GNG13). The enrichment of RNA metabolic activity in the significant proteomic dataset was represented by two big interconnected clusters associated with RNA processing and translation. Both clusters are linked to RNA-binding regulators involved in RNA transport and stability including FMRP, CAPRIN1, the members of Pumilio (PUM) and CUG-BP, Elav-like (CELF) family, and YTH N(6)-methyladenosine RNA-binding proteins (YTHDF).

The IPA network analysis on the individual RNA/DNA – protein or protein – protein interactions of FMRP with the other significant proteins connected FMRP to nuclear FMRP interactor – NUFIP2, RNA-binding regulator – PUM2, purine rich element binding protein – PURB, splicing factor – SFPQ, BCL2 binding anthanogene – BAG3 and tubulin alpha-4A chain – TUBAA. Additionally, the network showed close association to several signaling nodes of ERK1/2 and SRC family and to general regulation of gene expression through histones and RNA polymerase (**Figure 2B**).

## Discussion

The critical role of FMRP in hippocampus was previously demonstrated in human Fragile X syndrome (FXR) patients and mouse *FMR1* KO FXR models. A large spectrum of FXR-associated pathological changes found in mouse hippocampus include altered morphology of dendritic spines, behavioral changes, impairment of hippocampal synaptic plasticity and hippocampal-dependent memory tasks (reviewed in Bostrom et al., 2016). Furthemore, deficits in long-term memory in hippocampus and spatial learning were observed in rat models of FXR (Tian et al., 2017). Many of FXR symptoms share a high degree of similarity to systemic changes and cognitive decline during normal aging which points to aging-related changes in the processes regulated by FMRP.

The lack of FMRP in mouse and rat KO models have been previously associated with impaired functionality of DG and hippocampal-dependent spatial memory (Franklin et al., 2014; Hébert et al., 2014; Yau et al., 2016) with both affected during normal aging in mammals (Lister and Barnes, 2009). In the current study, FMRP levels were analyzed in the DG of young (17 weeks) and aging (22 months) rats and the results were confirmed by three different methodological approaches – quantitative proteomics, immunoblotting, and immunofluorescence microscopy. The cohorts of both animal groups were first behaviorally tested for performance in a hippocampal-dependent memory task and confirmed a significant decline in spatial reference memory in aging rats. Proteomic results showed a significant reduction of FMRP levels in the samples from the aging group. Immunoblotting was performed with KO-verified antibody MAB2160, previously used for analysis of FMRP in a rat FXR model (Till et al., 2015). Several isoforms of FMRP around 70 kDa were detected with all signals significantly decreased in the aging rats. Consistently with proteomic- and immunoblotting data, the reduction of FMRP levels in total area or individual subareas of aging DG was confirmed by immunofluorescence microscopy.

Reduction of FMRP in aging rats observed in the current study corroborates with the previous studies on changes of FMRP levels in total brain tissue from young and aging mice (Singh et al., 2007; Singh and Prasad, 2008). Interestingly, the authors found sex-related discrepancies in the level changes of *FMR1* mRNA with consistent reduction of FMRP in the aging group which was attributed to interactions with specific transcription factors (Singh and Prasad, 2008). All rats used in the experiments presented herein were males and the corresponding sex-related differences in the levels of FMRP and *FMR1* mRNA in the aging rat brain remain to be investigated.

In addition to FMRP the quantitative proteomic experiment allowed simultaneous detection of 153 proteins that were significantly up- or downregulated in the aging compared to young rats. Based on functional enrichment- and network analysis these proteins could be clustered into several groups implicated in the altered biological processes. The highest protein ratios were detected for proteoglycans and proteoglycan-associated proteins brevican (BCAN), neurocan (NCAN), versican (VCAN), aggrecan (ACAN), tenscin (TNC), and hyaluronan and proteoglycan link proteins (HAPLNs). These proteins are part of the extracellular brain matrix that plays a numerous roles in brain morphogenesis by controlling migration, proliferation and differentiation of neurons and facilitating molecular signaling (Oohashi et al., 2015). The experiments in KO mice proved that these molecules have an essential function in maintaining synaptic plasticity in the DG (Jansen et al., 2017). The proteins of membrane vesicles, lipid rafts and cell junctions formed another group significantly deregulated in the aging rats. These include Flotillins (FLOTs), Bridge integrator-1 (BIN1) or Protein Kinase C And Casein Kinase Substrate In Neurons 2 (PACSIN2) that were previously linked to synapse formation and function in hippocampus (Swanwick et al., 2010; Anggono et al., 2013; Monje et al., 2013; Zhang et al., 2015).

Proteins related to translation machinery and RNA processing were the most represented group among the dysregulated proteins. The synthesis of new proteins is a key element for basic cellular functions but has been also implicated more specifically in memory formation. It was shown that protein synthesis is important for synaptic remodeling underlying the long-term memory (Sutton and Schuman, 2006; Alvarez-Castelao and Schuman, 2015), involved in the consolidation phase of hippocampal-dependent learning (Rossato et al., 2007) and has a role in hippocampal synaptic plasticity (Cajigas et al., 2010). The pathway enrichment analysis from the current study revealed a significant downregulation of EIF2 signaling (z-score = 2.646) representing a decrease in protein synthesis in DGs of the aging group. This has been also reported by previous studies on young and aging Sprague-Dawley rats (Ingvar et al., 1985; Smith et al., 1995) and represents confirmation of the proteomic results. Interestingly, the study of Wei et al. (2015) recently revealed an important function of RNA-binding regulators in decoupling of transcription and translation processes during aging in prefrontal cortex of humans and monkeys. The network analysis of significant proteins presented herein connected the clusters of mRNA translation and splicing machineries to FMRP and several other RNA-binding regulators involved in RNA transport and stability including CAPRIN1, the members of Pumilio (PUM) and CUG-BP, Elav-like (CELF) family, and YTH N(6)-methyladenosine RNA-binding proteins (YTHDF). PUM proteins are closely related to FMRP through RNA-interactions and number of functions including DG neurogenesis and synaptic morphogenesis (Vessey et al., 2010; Zhang et al., 2017). CELF proteins has been proposed as regulators of synaptic plasticity and transmission in hippocampus (Wagnon et al., 2012).

## Conclusion

The study presented herein showed that the level of the functional synaptic regulator, FMRP, in the rat DG is significantly reduced during aging. In addition, quantitative proteomics and bioinformatics identified several dysregulated protein clusters. The results emphasized the role of FMRP and other RNA-binding regulators in the modulation of DG protein synthesis during aging that warrant further analysis. The complete list of dysregulated proteins obtained from current study is presented in the result section and supplementary material, and might provide a useful dataset for further studies on aging in the rat hippocampus.

## Author Contributions

Conceived and designed the experiments: RS, FS, VK, HH, JW, and GL; performing experiments: RS, MK, TS, DR, JM, DF, and JW; collected data and processed them: RS, FS, TS, JM, DF, DR, and VK; interpreted the results: RS, GL, DM, and JW; wrote paper: RS, GL, VK, and DR; revised the intellectual content: GL, VK, DM, and HH. All authors read and approved the manuscript.

## Conflict of Interest Statement

The authors declare that the research was conducted in the absence of any commercial or financial relationships that could be construed as a potential conflict of interest.

## Footnotes

1 https://string-db.org/

2 https://string-db.org/

## Supplementary Material

TABLE S1 The list of proteins identified in the quantitative proteomic study and the proteins with significantly changed levels between young and aging rats. The identified protein datasets with UniProt accession numbers, MS parameters and the statistics of the animal group comparison.

TABLE S2 The GO enrichment analysis and the identification of the most representative biological functions and canonical pathways associated with the differential protein dataset obtained from the quantitative proteomic study.
