# Supplementary material for: Reduced Levels of the Synaptic Functional Regulator FMRP in Dentate Gyrus of the Aging Sprague-Dawley Rat
Source: Front Aging Neurosci. 2017 Nov 23;9:384. doi: 10.3389/fnagi.2017.00384 (PMC5703695; doi:10.3389/fnagi.2017.00384)
Supplement: Supplementary file 3 [file Image_1.PDF]

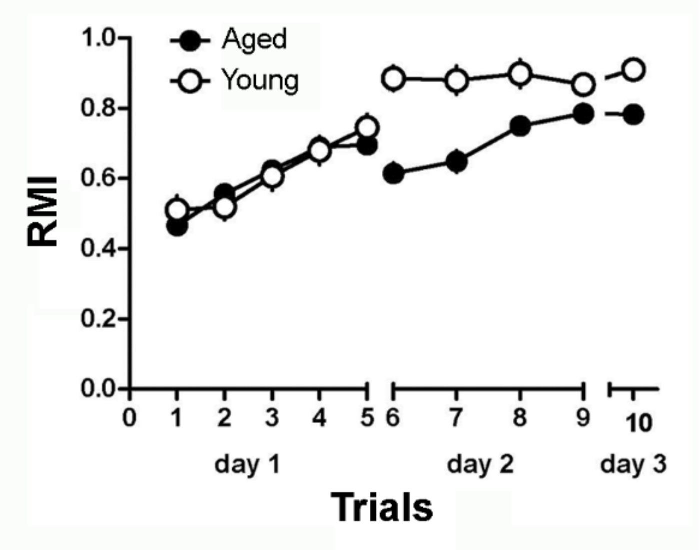

Figure S1: Behavioral testing of the cohort of young and aging animals used for quantitative proteomics. Performance of young and aged rats in the hole-board memory task across three consecutive days. Reference Memory Index (RMI) was calculated using the formula (first + revisits of baited holes) / total visits of all holes. The values are expressed as mean ± SEM. Significant differences between two groups were observed at day 2 ($p < 0.05$) and at the retention test at day 3 ($p < 0.05$) with better performance of young animals.
