# Supplementary material for: Reduced Levels of the Synaptic Functional Regulator FMRP in Dentate Gyrus of the Aging Sprague-Dawley Rat
Source: Front Aging Neurosci. 2017 Nov 23;9:384. doi: 10.3389/fnagi.2017.00384 (PMC5703695; doi:10.3389/fnagi.2017.00384)
Supplement: Supplementary file 4 [file Image_2.PDF]

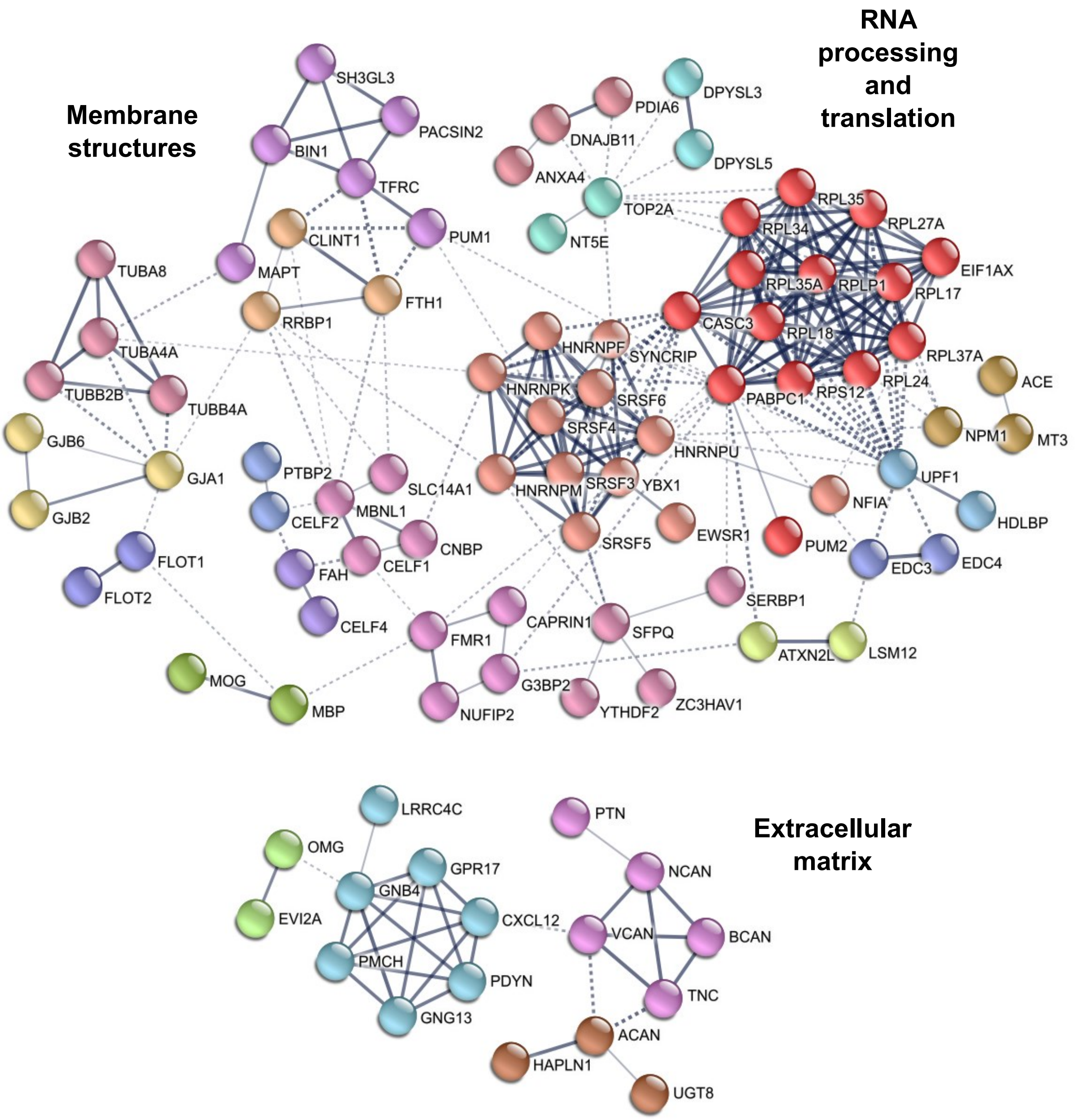

Figure S2: The interaction network of the significantly changed proteins obtained by STRING v10.5 clustered by MCL algorithm (https://algorithm string-db.org). The groups of functionally closely related proteins include clusters linked to formation and transport of membrane structures, RNA processing and extracellular matrix. The confidence of identified interactions between the nodes is given by thickness of the edges. Full lines represent the network clusters, the dashed lines represent the connections between the clusters. The parameters of the analysis were set to confidence threshold 0.5 and inflation parameter 3.
